# The Role of Orthodontics in the Management of Maxillofacial Fractures in Children: A Review on Contemporary Approaches

**DOI:** 10.7759/cureus.63128

**Published:** 2024-06-25

**Authors:** Manaf O Alhabshi, Duaa M Taweel, Hashm M Alahmary, Osama H Al-Suhaymi, Mohammed R Al-Bander, Taif A Al-Suroor, Afaf M Al-Shahrani, Bayan H Alshallaa, Bushra A Bakhamis

**Affiliations:** 1 Oral and Maxillofacial Surgery, King Abdullah Medical City, Jeddah, SAU; 2 Dentistry, Ministry of Health, Jeddah, SAU; 3 Faculty of Dentistry, Umm Al-Qura University, Makkah, SAU; 4 Dentistry, Taibah University, Madinah, SAU; 5 Dentistry, Imam Abdulrahman Bin Faisal University, Dammam, SAU; 6 Dentistry, Ministry of Health, Abha, SAU; 7 General Dentistry, Ministry of Health, Al-Ahsa, SAU; 8 General Dentistry, Armed Forces Hospitals Administration in Taif Region, Taif, SAU

**Keywords:** maxillofacial trauma, pediatric maxillofacial surgery, pediatric facial trauma, ortho-surgery, pediatric fractures, maxillofacial fracture

## Abstract

Maxillofacial fractures present complex challenges requiring effective orthodontic management to restore function and aesthetics. This review explores various orthodontic techniques, including fixed braces, maxillomandibular fixation (MMF), functional orthodontic therapy (FOT), and acrylic splints, emphasizing their roles in stabilizing fractures and promoting healing. The management of condylar fractures is discussed, highlighting the benefits of early intervention with functional appliances to facilitate condylar remodeling in children and adolescents. Additionally, the review covers splinting methods for dental and dentoalveolar fractures and the use of open reduction internal fixation (ORIF) for maxillary fractures. It addresses the complications and challenges of fracture management, the need for a multidisciplinary approach, and the limitations of current studies. Future directions include the use of advanced technologies such as virtual surgical planning (VSP) and 3D printing to enhance treatment precision and outcomes. This review provides a comprehensive overview of orthodontic strategies for maxillofacial fractures, offering insights into clinical applications and future advancements.

## Introduction and background

Orthodontics plays a very important role in the management of maxillofacial fractures in growing children; it restores anatomy, occlusion, and aesthetics of the facial complex; eliminates deformity; restores masticatory abilities; and enables early return to function [1]. When it comes to treating maxillofacial fractures, the modalities differ depending on the type of jaws involved and whether the fractured segments are displaced or undisplaced. For displaced fractures, open reduction internal fixation (ORIF) is recommended for achieving anatomic reduction [2]. However, this method of fracture reduction carries the risk of post-operative infection. It is also not recommended in children for several reasons, such as the potential risks of visible scarring, nerve injury, and disturbances in post-surgical growth patterns [3].

In undisplaced fractures, the usual treatment approach is maxillomandibular fixation (MMF), which can be implemented using Erich arch bars, passive arch wires, or screws [4,5]. However, this method of reduction also has several disadvantages, including difficulty eating and maintaining oral hygiene, post-operative discomfort, damage to periodontal tissues, and enamel demineralization [6].

ORIF and MMF are the usual management of choice in adults. However, the management of maxillofacial fractures in children poses a unique challenge to maxillofacial surgeons. Furthermore, fractures involving both facial bones and dentition require a multidisciplinary approach for optimal outcomes [5]. There are several reasons why the management of maxillofacial fractures differs in children as compared to adults. First, fractures in children tend to be greenstick fractures with minimal or no displacement [7-9]. Not only that, children have better osteogenic potential with very rare chances of fibrous/non-union. Therefore, even if the fracture is imperfectly reduced, it has a better chance of healing correctly [10]. Next, the mandible in children is a growing bone that incorporates developing tooth buds. Attempts made to fix mandibular fractures with rigid plates could potentially hamper the growth of the mandible and therefore risk damaging tooth buds [11,12]. Moreover, the use of rigid arch bars is not indicated in children for several reasons. Deciduous teeth are conical in shape with resorbing roots. Conversely, permanent teeth that have erupted in the oral cavity have incomplete roots. Therefore, they cannot provide sufficient anchorage for arch bar fixation [13].

Given these factors, orthodontic management of certain types of maxillofacial fractures requires a more conservative and less invasive approach. Orthodontic intervention encompasses a spectrum of strategies for addressing trauma-induced malocclusion, facial asymmetries, and associated soft tissue injuries. The technique involves realigning dentition and stabilizing occlusion as well as fractured segments [14-16]. These techniques harness the natural osteogenic abilities of the bone to enhance healing, thus facilitating fracture reduction and stabilization [14].

This article aims to provide a comprehensive overview of the various orthodontic techniques used in the management of facial fractures in pediatric and adolescent patients. In the management of facial fractures, particularly maxillary fractures, it is important to note that certain types of fractures, such as Le Forte fractures, often require open reduction and internal fixation and are not typically treatable by orthodontic means. These fractures typically involve significant displacement of the maxilla, and surgical intervention is needed to restore proper anatomical alignment and function. However, dentoalveolar fractures are amenable to orthodontic management, often in conjunction with conservative or surgical approaches. While this article primarily addresses mandibular fractures and maxillary dentoalveolar fractures, it does not cover the management of complex maxillary fractures that require open reduction and internal fixation.

By analyzing existing literature and discussing clinical approaches, this article seeks to fill gaps in current knowledge and serve as a valuable resource for orthodontists, oral, and maxillofacial surgeons and other healthcare professionals involved in the care of patients with facial fractures. Additionally, this article aims to contribute to improved patient outcomes and enhanced understanding of the role of orthodontics in facial fracture management by highlighting the importance of interdisciplinary collaboration and tailored treatment approaches. Ultimately, the goal is to empower clinicians with the knowledge and tools needed to effectively address the complex challenges associated with the orthodontic management of facial fractures, thereby optimizing patient care and enhancing overall treatment success.

## Review

Article selection

The article selection process involved searching electronic databases, including PubMed, Medline, and Google Scholar, using relevant keywords such as “orthodontic management,” “functional treatment,” “maxillofacial fractures,” “mandibular fractures,” “condylar fractures,” “pediatric,” “children,” and “adolescent”. The initial screening was based on title and abstract relevance, followed by full-text reviews of potentially eligible articles. Articles that addressed orthodontic treatment options for maxillofacial fractures in pediatric or adolescent populations were included. The final selection was based on relevance to the research objectives, methodological quality, and contribution to the research topic.

The articles or studies that fulfilled the following criteria were included: articles published in English with no timeline restriction, studies investigating orthodontic management of maxillofacial fractures in human subjects; studies that included both pediatric populations; studies with a clear description of fracture types, treatment protocols, and outcomes; studies that involved various orthodontic treatment modalities, including but not limited to brackets, orthodontic appliances, functional therapy and splints; studies that provided detailed information about entire treatment protocol including appliance selection, duration of the treatment, and follow up; and finally retrospective studies, case reports, and systematic reviews that were accessible through peer-reviewed journals.

The studies were not included if they fulfilled the following criteria: animal studies, in vitro studies, studies done on adult patients, studies focusing only on surgical management of maxillofacial fractures without any kind of orthodontic intervention, studies with inadequate methodologies, studies that lacked clear documentation of orthodontic treatment protocol, studies that did not involve follow-up, and studies which were not accessible through peer-reviewed journals.

Different orthodontic techniques and appliances used for the treatment of maxillofacial fractures

Orthodontic Brackets and Wires

Various orthodontic techniques are employed in the management of facial fractures to restore occlusal harmony, facial aesthetics, and function. One commonly utilized technique involves using orthodontic appliances such as fixed braces to reposition displaced teeth and stabilize dental arches following fracture reduction [14,15]. These appliances apply controlled forces to guide teeth into their proper alignment and ensure proper occlusion. The use of orthodontic brackets for MMF has several advantages over Erich arch bars. The pain-free method does not require anesthesia or wide mouth opening, and it has better patient compliance for maintaining oral hygiene. In addition, application as well as removal of the orthodontic brackets is convenient for the patient and the orthodontist [14,15]. A prospective clinical study was conducted on 20 patients who suffered from multiple facial fractures. The treatment protocol included MMF with or without ORIF. For MMF, orthodontic brackets were used instead of Erich arch bars. The brackets were positioned accordingly considering the pre-traumatic occlusion. Niti wires were then secured in brackets with modules, and MMF was done using ligature wires around the upper and lower bracket hooks for the stabilization of fractured segments. Where indicated, elastics were used to bring teeth to proper occlusion. The duration of MMF in patients without ORIF was two to six weeks, and MMF was kept for two to three weeks in patients who needed ORIF. The brackets were removed at the end of the sixth week. The clinical follow-up was done at two to three days, one-month, three-month, and six-month intervals along with an orthopantomogram and CBCT if indicated. IMF with orthodontic brackets had satisfactory outcomes with better patient convenience and proper bone healing [16].

Functional Orthodontic Therapy (FOT)

FOT may also be employed to address skeletal discrepancies and malocclusion resulting from facial fractures. This approach utilizes appliances such as functional appliances to correct underlying skeletal issues and improve facial symmetry and function. FOT is usually indicated in growing patients with mandibular condylar fracture, and condylar remodeling following functional appliance use is thought to be the primary mode of healing. A prospective study was conducted on 55 children between 2 1/2 and 9 3/4 years of age who presented with a unilateral fracture of the mandibular condyle. The patients were treated conservatively using a myofunctional appliance, i.e., activators. The follow-up included standardized clinical examination and evaluation with panoramic radiographs at six, 12, 24, 48, and 72 weeks post-trauma. The clinical outcomes were satisfactory with no functional disturbance or facial asymmetry, and the condyles of 47 out of 55 patients were in fairly good shape. While functional therapy aims to intervene early and address issues non-surgically, the stabilization of the TMJ over time necessitates a more comprehensive surgical solution to achieve lasting correction of malocclusion and restore optimal function and aesthetics to the jaw [17].

Acrylic Splints

Acrylic splints offer a versatile treatment avenue for juvenile complex facial traumatic fractures, and they restore both function and aesthetics while minimizing associated morbidity. Importantly, they do not hinder jaw growth or dentition development, making them suitable for patients of various ages [18]. The fractured segments are first reduced on the cast of the patients by splitting the cast into two parts and then realigning them in a normal position. The splints are then fabricated using acrylic and secured on the jaws of patients using circum-mandibular wiring. Additionally, the splints are reinforced with the incorporation of orthodontic wires. Splints have various advantages, including ease of application and removal, expedited construction, reduced operating times, optimal stability during healing, minimal impact on adjacent anatomical structures, and facilitation of tooth-to-tooth contact to aid in occlusion-guided reduction [13].

Furthermore, occlusal splints combined with circum-mandibular wiring in displaced fractures present additional benefits, such as applicability across diverse age groups, early mobilization, and lowered risks of muscular atrophy or ankylosis [19]. A previous study examined a five-year-old male who presented with upper and lower jaw injuries from a fall. Clinical examination revealed facial asymmetry, sublingual hematoma, and lip laceration. Radiographic imaging confirmed bilateral condylar fractures, symphyseal fractures, and a Le Fort II fracture on the right. Preoperative orthopantomography and computed tomography scans further delineated the extent of the fractures. For this patient, open cap splints were fabricated using alginate impressions and dental stone casts, and arch bar hooks were incorporated for intermaxillary fixation (IMF). Molar tubes were placed in the maxillary splint for inter-zygomatic wiring. This multidisciplinary approach aimed to stabilize fractures and promote optimal recovery. Postoperative follow-up was done weekly for two weeks, and uneventful healing was noted. The IMF elastics were removed after 10 days, while the open cap splint was kept in place. The splint was removed under local anesthesia after two months due to missed follow-up, revealing stable occlusion and good healing on the orthopantomogram. Monthly follow-up continued for five months, with a favorable prognosis [20].

Splinting of Teeth

Following maxillofacial fractures, dental and dentoalveolar fractures are the most common findings in children and adolescents, depending upon the intensity of the fracture. For these types of fractures, splinting the teeth is the recommended treatment option. A splint is defined as “an apparatus used to support, protect or immobilize teeth that have been loosened, replanted, fractured or subjected to certain endodontic procedures.” [21] When it was first used, splinting was very rigid and involved long-term immobilization, which showed an increased incidence of pulp necrosis and external root resorption of teeth [22-24]. Later on, the International Association of Dental Traumatology (IADT) recommended flexible splints for short durations to provide functional movements to the traumatized teeth [25]. This method of stabilizing the fractured dentoalveolar segments utilizes orthodontic wires and composite resin. As a type of non-rigid (flexible) splinting, it has several advantages including allowing for physiologic tooth mobility, ease of maintaining oral hygiene, and improved patient comfort.

Overall, the selection of orthodontic techniques in facial fracture management depends on various factors, including fracture severity, patient age, skeletal maturity, and treatment goals. Orthodontists can effectively restore dental and facial harmony in patients with facial fractures by using a personalized approach that integrates orthodontic principles with surgical and conservative modalities, thereby enhancing their quality of life and functional outcomes.

The following sections of the article provide comprehensive insights into the orthodontic management of individual fractures and offer detailed explanations of specific treatment approaches tailored to each type of fracture.

Orthodontic management of condylar fractures

Orthodontic Management of Condylar Fractures

The mandibular condyle is the most common site of facial injury in children as well as adults [26]. In patients who have experienced trauma, the timing of treatment plays a crucial role in determining the most appropriate course of action. Functional therapy is a viable option within the first three months after the traumatic event. This therapy focuses on restoring proper function to the temporomandibular joint (TMJ) and addressing any resultant malocclusion. However, as time progresses beyond the initial three-month window and exceeds six months post-trauma, the dynamics shift. At this stage, the TMJ typically undergoes a process of stabilization, accompanied by the development of malocclusion. In such cases, orthognathic surgery becomes the primary treatment option. [27] This shift in treatment approach reflects the evolving nature of post-traumatic TMJ conditions.

In children and adolescents, condylar fractures account for 72-80% of all mandibular fractures [28]. Boys are more commonly affected than girls [29]. Due to highly vascularized condylar heads, significant amounts of medullary bone, and thin cortices, condylar fractures are more prevalent in children than symphyseal or parasymphyseal fractures [30]. Furthermore, condylar fractures are commonly overlooked and left undetected because they are almost always caused by indirect trauma [31]. These undiagnosed condylar fractures in children later manifest as disturbances in mandibular growth, facial asymmetry, ankylosis, malocclusion, and pain either on the same or both sides. In growing children, mandibular condyle fractures can lead to TMJ ankylosis and dysfunction along with disturbed mandibular growth [32]. Surgical management of fractured condyles is not typically indicated in children due to the risks of scarring, nerve damage, and impaired mandibular growth [3,33]. However, conservative treatment with or without IMF can potentially cause late complications, such as ankylosis, disturbed facial growth, and functional TMJ disorders [34-36]. Orthopedic treatment with functional appliances used for the treatment of class II malocclusion due to retrognathic mandible remains the choice of treatment in children because of its association with the remodeling of the condylar head and glenoid fossa [37,38]. Remodelling of the fractured condyle after functional appliance use, however, depends on the age of the patient. In patients up to 10 years of age, the development of the new condyle is more probable, and there is a better chance of initial remodeling [33]. Conversely, orthodontic appliances with loose fittings, such as the monobloc, may promote activation of the protractor and elevator muscles to maintain their position. This potentially makes them advantageous in orthopedic rehabilitation for condylar fractures [39]. To evaluate the conservative treatment approach, researchers conducted a retrospective magnetic resonance imaging (MRI) study in patients less than 18 years of age using a functional orthodontic appliance (FOT), i.e., a spring activator. Along with clinical and functional findings, the follow-up was done with MRI scans. None of the patients reported functional limitations and alterations in occlusal relationships. The FOT led to favorable functional and morphologic outcomes. The follow-up with MRI demonstrated physiological remodeling of the condyle with proper restitution of ramus height [40]. Another study evaluated the developmental and functional outcomes of unilateral condylar fractures in eight children and adolescents between 5 and 13 years of age treated with removable occlusal splints. The thickness of the splint was determined by the age of the patient, stage of dentition, and level and degree of dislocation of condylar fracture. The patients were asked to wear the splint for one to three months, along with functional exercises. The follow-up included clinical observation, orthopantomogram, TMJ cone beam computed tomography, and electromyography of masticatory muscles. The follow-up visits were planned at one, three, and six months after treatment and then annually. The clinical examination was done at each visit, and the radiological examination at six months and annually. All the patients showed satisfactory results [41]. A retrospective study was conducted among 40 children and adolescents between three and 16 years of age with condylar fractures. All participants were given removable acrylic splints of varying thickness depending on the age of the patient, the developing stage of the mandible, and the degree to which the condyle was dislocated. The patients were asked to wear the splints for one to three months accompanied with functional exercises for more than six months. The clinical follow-up demonstrated stable occlusion with normal growth, development, and function of the mandible, whereas the orthopantomogram showed physiologic remodeling and reconstruction of the fractured condyles [42].

A 10-year and nine-month-old male patient who had bilateral condylar and symphyseal fractures in a car accident was treated conservatively with orthodontic brackets and elastics. Brackets (0.022-inch slots) were placed on specific teeth without local anesthesia or sedation. A 0.018-inch Australian wire was customized to match the bracket level, with ligature wire loops or hooks used for elastic wear. Intermaxillary elastics were applied from the maxillary to the mandibular teeth. Four rubber bands per side ensured secure IMF, and they were changed daily for four weeks. The patient adhered to a liquid diet for the initial two weeks and avoided mouth opening, except for cleaning purposes [43].

Orthodontic management of mandibular body fractures

The mandible is a unique U-shaped bone that articulates with the cranial base at the TMJ [44]. Mandibular fractures contribute 10-25% of all facial injuries, the most common etiology being road traffic accidents or interpersonal attacks [45]. Post-traumatic occlusion is the most important factor that will determine whether the fracture can be handled conservatively or requires surgical intervention [46].

Rigid acrylic splints are usually indicated in symphyseal, parasymphyseal, or body fractures of the mandible to promote healing and stabilization [19]. These could either be open or closed splints. Open splints are indicated in case of displaced fractures that need to be stabilized with the help of circum-mandibular wiring [20]. Conversely, closed occlusal splints can be directly cemented over teeth in minimally or completely undisplaced fracture segments [47]. A previous case study documented the conservative management of displaced mandibular body fractures in children with open acrylic splints. In all the patients, the fractured segments were reduced and conservatively stabilized with an open acrylic splint secured with circum-mandibular wiring. The splints were removed after three weeks with successful outcomes [48]. In a different case report, a five-year-old child with a parasymphyseal fracture was treated with orthodontic splints. The follow-up after 10 years showed stable results [49]. In another case report of symphyseal fracture with minimal displacement, the rigid cemented acrylic splint was reinforced with 0.5 mm stainless steel wire between mandibular incisors [50]. An interesting case of a 10-year-old boy, who had bilateral condylar fractures with symphyseal fracture, was published. The treatment protocol included bonding of orthodontic brackets with 0.018 Australian wire, and elastics were used for the stabilization of fractured segments [43]. The recent advances in the management of facial fractures include virtual surgical planning and 3D printing. The preoperative 3D design of the 3D-printed splint facilitates surgical fracture reduction, enhancing operative precision. Additionally, splints that are tailored to the child's dentition offer superior stability compared to traditional dental arch splint ligation. Furthermore, by obviating intraoperative impression-taking and the fabrication of occlusal pads/retainers, it minimizes contamination risks in the surgical area, streamlines operative procedures, and enhances safety and efficiency [51]. In a case report of a child with a fractured mandible, virtual surgical planning using ProPlan CMF 1.4 software enabled precise fracture segment repositioning and anatomical simulation. Subsequently, Geomagic Studio 6.0 software was used to design the occlusal splint based on stereolithography data of the reconstructed mandible model. The splint was fabricated using a 3D printer (3D System ProJet3510s), ensuring accurate reproduction of the lower dentition [52]. In a child with symphyseal and bilateral condylar fracture, a 3D-printed occlusal splint was utilized to ensure accurate mandibular repositioning and fixed with bilateral MMF screws. The splint was positioned on the maxillary dentition and secured to the screws with wire loops, establishing a reference basis for mandibular adduction. An absorbable plate, contoured to the restored model, was affixed at the fracture site. Postoperative imaging confirmed successful mandibular adduction, with favorable outcomes observed during the two-month follow-up period. This approach is particularly beneficial among children with mandibular symphyseal fractures accompanied by bilateral condylar fractures [53].

Orthodontic management of dental and dentoalveolar fractures

As mentioned above, dental and dentoalveolar injuries are the most frequently occurring injuries after maxillofacial fractures; they usually occur due to falls, contact sports, and accidents [54]. The commonly affected teeth are maxillary central incisors [55], followed by maxillary lateral incisors and mandibular incisors [56], especially in children with proclined dental and dentoalveolar segments [57]. The International Association of Dental Traumatology (IADT) has recommended different splinting times for different types of dental injuries. For subluxation, extrusive luxation, and avulsion, the splinting time is two weeks. On the other hand, for lateral luxation, intrusive luxation, root fracture, avulsion with a dry time of more than 60 minutes, and dentoalveolar fractures, the splinting time should be four weeks [25].

There are different types of splints currently in use, such as composite and wire splints, orthodontic brackets and wire splints, fiber splints, titanium trauma splints, arch bar splints, etc. In clinical practice, the most commonly used splints are composite and wire splints. The diameter of the wire used should not be more than 0.3-0.4 mm [58]. In orthodontic brackets and wire splints, brackets are bonded to the teeth using bonding agents, and a 0.014” Niti flexible wire is connected [59]. The other type of splints is fiber splints, which utilize polyethylene or Kevlar fiber mesh to stabilize the fractured teeth. These are directly bonded to the tooth surface. Fiber splints are found to have the highest incidence of favorable healing [60]. Titanium-fibre splints, developed by von Arc, are made up of 0.2-mm-thick and 2.8-mm-wide titanium. Its rhomboid mesh structure is used to secure the splints on the tooth structure [61]. Arch bar splints are usually recommended in dentoalveolar fractures. These splints are made of metal arch bars fixed over teeth with the help of ligature wires.

In a previous case report, a 14-year-old female patient had a dentoalveolar fracture with traumatic intrusion of maxillary left central and lateral incisors. The patient was treated conservatively by repositioning the displaced incisors to occlusion and splinting them with a 21x25 stainless steel orthodontic archwire. This repositioning kept the incisors and the fractured dentoalveolar segments stable and allowed primary healing of the bone. A clinical and radiographic follow-up after six months showed satisfactory outcomes. The incisors were later treated with root canal treatment [62].

Another case report of a nine-year-old boy discussed traumatic intrusion of maxillary incisors with dentoalveolar fracture of the anterior maxilla. In this patient, the intruded teeth and fractured alveolar segments were replaced under local anesthesia, and stabilization was done by wire splinting technique with composite resin. The splint was removed after four weeks per the guidelines of the International Association of Dental Traumatology and the American Association of Endodontists. In the follow-up visits, the patient had asymptomatic teeth with intact lamina dura and satisfactory bone healing [63]. A case series was published where different types of splints were utilized in adolescent patients for the management of traumatic injuries to anterior teeth [64].

Orthodontic management of maxillary fractures

Facial trauma involving maxillary segments is a rare injury accounting for 0.5-2% and poses a unique challenge to surgeons. Maxillary injuries are reported to be common emergencies in acute settings caused by road traffic accidents (RTA), interpersonal assaults, or sports [65]. Due to its close proximity to important anatomic vital structures including the brain, urgent assessment and treatment are mandatory to avoid morbidity and mortality. ORIF is the most common treatment option for maxillary fractures because of its stable and precise anatomic reduction [66]. Conservative treatment is indicated only in minimally and undisplaced fractures, and it includes analgesia, a soft diet, and avoiding further trauma.

Complications and challenges

The complexity of fractures, which can vary widely in severity and extent, poses a treatment challenge; thorough assessment and treatment planning are needed to achieve stable occlusion and facial harmony. Additionally, the risk of interference with the healing process and potential complications, such as root resorption or TMJ dysfunction, underscores the importance of cautious and informed decision-making. Patient cooperation can also be challenging, particularly in cases where pain, discomfort, or difficulty with oral hygiene may affect treatment adherence. Furthermore, a multidisciplinary approach is needed to achieve long-term stability and optimal outcomes in the orthodontic management of maxillofacial fractures; this involves close collaboration between orthodontists, oral and maxillofacial surgeons, and other specialists. Addressing these challenges requires a combination of clinical expertise, interdisciplinary collaboration, and patient-centered care to optimize treatment success and improve patient outcomes in the orthodontic management of maxillofacial fractures.

Limitations and future directions

The studies done for the evaluation of orthodontic management of maxillofacial fractures have limited sample sizes as most of the literature is case reports, which may have predominantly included cases with desirable outcomes, excluding more complex cases. This provides anecdotal evidence rather than scientific evidence, limiting its strength. It may lead to an incomplete understanding of the efficacy of orthodontic treatment in maxillofacial fractures.

There is also a lack of comparative studies evaluating the outcomes of orthodontic intervention with that of surgical management or a conservative approach. Another evident limitation was the lack of standardization of study design across different studies, including methodologies, outcomes, and follow-up periods. This makes it challenging to compare the results.

There is a need to address these limitations through well-designed prospective studies with categorized sample sizes, standardized methods, and longer follow-up periods. This will help enhance our understanding of orthodontic management of maxillofacial fractures and improve clinical practice guidelines.

As mentioned in the limitations, conducting prospective clinical trials with larger sample sizes across different age groups can help evaluate the efficacy and long-term outcomes of the conservative orthodontic approach. Similarly, conducting biomechanical studies can further elucidate the magnitude of forces involved in the orthodontic treatment of maxillofacial fractures. This will in turn help researchers optimize the treatment protocols for predictable outcomes. In addition, research that prioritizes patient-centered outcomes can help in assessing various factors such as patient satisfaction, quality of life, and psychological effects post-treatment. Finally, integrating various recent technologies, such as virtual surgical planning (VSP), computer-aided design, computer-aided manufacturing (CAD-CAM), and 3D printing, can improve the precision and efficiency of the orthodontic approach in the management of maxillofacial fractures.

## Conclusions

Orthodontics plays a crucial role in managing maxillofacial fractures by addressing occlusal discrepancies, dental injuries, facial asymmetries, and temporomandibular joint issues, thus contributing to functional and aesthetic restoration. Orthodontists collaborate closely with oral and maxillofacial surgeons to develop tailored treatment plans that ensure optimal outcomes and enhance patients' quality of life. However, there is a need for high-quality research, including prospective clinical trials with larger sample sizes and long-term follow-up, to further advance this field.

Emerging technologies such as CAD-CAM and 3D/4D printing offer opportunities for precise treatment planning and customization. Additionally, advancements in tissue engineering and biomaterials hold promise for improved bone healing and soft tissue regeneration. By embracing these innovations and fostering interdisciplinary collaboration, orthodontists can continue to enhance treatment options and outcomes for patients with maxillofacial fractures.
